# Human subthalamic nucleus–medial frontal cortex theta phase coherence is involved in conflict and error related cortical monitoring

**DOI:** 10.1016/j.neuroimage.2016.05.031

**Published:** 2016-08-15

**Authors:** Baltazar Zavala, Huiling Tan, Keyoumars Ashkan, Thomas Foltynie, Patricia Limousin, Ludvic Zrinzo, Kareem Zaghloul, Peter Brown

**Affiliations:** a Nuffield Department of Clinical Neurology, University of Oxford John Radcliffe Hospital, Oxford, OX3 9DU, UK; b Surgical Neurology Branch, National Institutes of Health, 10 Center Drive, 3D20, Bethesda, MD 20814, USA; c Medical Research Council Brain Network Dynamics Unit at the University of Oxford, Mansfield Road, OX1 3TH, UK; d Department of Neurosurgery, King's College Hospital, King's College, London SE5 9RS, UK; e Sobell Department of Motor Neuroscience & Movement Disorders, UCL Institute of Neurology, London WC1 3BG, UK

**Keywords:** DBS, deep brain stimulation, DLPFC, dorsolateral prefrontal cortex, ITPC, intertrial phase consistency, LFP, Local Field Potential, mPFC, medial prefrontal cortex, PD, Parkinson's disease, STN, subthalamic nucleus, UPDRS, Unified Parkinson's Disease Rating Scale, Conflict, Subthalamic nucleus, Theta, Coherence, Errors

## Abstract

The medial prefrontal cortex (mPFC) is thought to control the shift from automatic to controlled action selection when conflict is present or when mistakes have been recently committed. Growing evidence suggests that this process involves frequency specific communication in the theta (4–8 Hz) band between the mPFC and the subthalamic nucleus (STN), which is the main target of deep brain stimulation (DBS) for Parkinson's disease. Key in this hypothesis is the finding that DBS can lead to impulsivity by disrupting the correlation between higher mPFC oscillations and slower reaction times during conflict. In order to test whether theta band coherence between the mPFC and the STN underlies adjustments to conflict and to errors, we simultaneously recorded mPFC and STN electrophysiological activity while DBS patients performed an arrowed flanker task. These recordings revealed higher theta phase coherence between the two sites during the high conflict trials relative to the low conflict trials. These differences were observed soon after conflicting arrows were displayed, but before a response was executed. Furthermore, trials that occurred after an error was committed showed higher phase coherence relative to trials that followed a correct trial, suggesting that mPFC–STN connectivity may also play a role in error related adjustments in behavior. Interestingly, the phase coherence we observed occurred before increases in theta power, implying that the theta phase and power may influence behavior at separate times during cortical monitoring. Finally, we showed that pre-stimulus differences in STN theta power were related to the reaction time on a given trial, which may help adjust behavior based on the probability of observing conflict during a task.

## Highlights

• During conflict STN theta phase synchronizes with that of the mesial frontal cortex.
• The increased synchrony is associated with stimulus induced phase locking.
• Increased synchrony was also observed in trials that followed an error.
• Pre-stimulus STN theta power correlated with reaction time but only during conflict.

## Introduction

Being able to execute tasks quickly and accurately is a key skill. Equally as important, however, is the ability to dynamically alter the amount of time dedicated to a task based on the task's difficulty and based on previous performance. One area of the brain implicated in speed-accuracy trade off, particularly in scenarios that require a quick action in the face of conflict, is the medial prefrontal cortex (mPFC). The mPFC is thought to include areas implicated in cognitive control such as the anterior cingulate cortex and the pre-supplementary motor area (Botvinick et al., 2001, Botvinick et al., 2004, Ridderinkhof et al., 2004). Previous work has shown that mPFC activity is not only higher for high conflict tasks (Botvinick et al., 2004, Sheth et al., 2012) it is also directly correlated to the reaction time during conflict (Cavanagh et al., 2011, Cohen and Cavanagh, 2011, Cohen and Donner, 2013). According to some models (Cavanagh et al., 2011, Cavanagh et al., 2012, Cohen and Cavanagh, 2011), increased mPFC theta band (4–8 Hz) activity is thought to increase the amount of evidence that has to be integrated by the brain prior to the selection of an action (i.e. increased “evidence threshold”). The mechanisms by which the mPFC is able to rapidly and dynamically alter behavior, however, are still unclear.

There is growing evidence suggesting that rapidly conducting hyperdirect inputs from the mPFC to the subthalamic nucleus (STN) allow the mPFC to inhibit the activity of motor networks and thus increase the amount of evidence that is needed to select an action during conflict (Zavala et al., 2015b). Both the subthalamic nucleus (STN) (Fumagalli et al., 2011, Brittain et al., 2012, Zavala et al., 2013, Zavala et al., 2014, Zavala et al., 2015a) and the mPFC (Cavanagh et al., 2011, Cavanagh et al., 2012, Cohen and Cavanagh, 2011) show similar increases in theta band activity during conflict. Furthermore, deep brain stimulation of the STN for Parkinson's diseases disrupts the relationship between increases in mPFC theta activity and increases in “evidence threshold” (Cavanagh et al., 2011, Ratcliff and Frank, 2012). The result of DBS related disruption of the network is thus rapid, impulsive actions with decreased accuracy (Frank et al., 2007, Coulthard et al., 2012, Green et al., 2013). Recently, a direct link between mPFC and STN oscillatory activity was established as the theta activity of the mPFC was shown to drive that of the STN in a dot motion discrimination task that involved gradual increases in conflict (Zavala et al., 2014). Whether or not this mechanism is also involved during abrupt onsets of conflict, as well as how the phase coherence between the two sites relates to the theta phase resets that are associated with rapid stimulus onsets (Cavanagh et al., 2012, Zavala et al., 2013), remains unknown.

Another outstanding question centers around the important role the mPFC seems to play in across-trial adaptations to the level of conflict or to errors (Kerns et al., 2004, Ridderinkhof et al., 2004, Danielmeier and Ullsperger, 2011). mPFC theta activity seems to interact with areas of the dorsolateral prefrontal cortex (DLPFC) following high conflict and error trials, which may be related to the respective speeding and slowing of reaction times on subsequent trials (Kerns et al., 2004, Hanslmayr et al., 2007, Cavanagh et al., 2009). Whether or not these interactions also involve the STN remains an open question, but a link is suggested by studies showing error related activity in the STN (Brown et al., 2006, Zavala et al., 2013, Bastin et al., 2014, Cavanagh et al., 2014, Siegert et al., 2014).

In this study, we build on our previous work in which we have separately shown that the flanker task induces theta band phase locking which entrains individual STN neurons (Zavala et al., 2013, Zavala et al., 2015a) and that mesial frontal cortex theta oscillations drive those of the STN in a gradually evolving conflict task that did not involve stimulus locked theta phase locking (Zavala et al., 2014). Specifically, here we investigate whether theta phase locking between mesial frontal cortex and STN also occurs in tasks in which conflict onset is abrupt and whether any activities in the STN correlate with across trial adaptations to conflict and errors.

## Methods

### Subjects and task

Thirteen subjects (13 males; mean disease duration, 10 years; mean age, 56 years; age range, 42–69 years) were recruited at the Oxford University Hospitals Trust, University College London Hospital, and King's College Hospital. All subjects gave their written informed consent to take part in the study, which was approved by the National Research Ethics Service Oxfordshire Rec A Committee. All subjects underwent bilateral implantation of DBS electrodes into the STN, as a prelude to high-frequency stimulation for the treatment of advanced PD. Only two patients had been diagnosed as having an impulse control disorder by their supervising neurologist, as documented in their hospital notes. One of these two patients was excluded for reasons discussed below. The other patient had behavior well within the range of that seen by the other patients and was therefore included in the study. Techniques to target and implant electrodes in the STN have previously been described (Foltynie and Hariz, 2010). Lead location was confirmed with intraoperative stereotactic MRI at University College London Hospital and with immediate postoperative stereotactic computed tomography at the remaining centers. Effective stimulation was confirmed intraoperatively. The permanent quadripolar electrode used was model 3389 (Medtronic Inc., Minneapolis, USA) featuring four cylindrical contacts. Electrode extension cables were externalized through the scalp to enable recordings before connection to a subcutaneous DBS pacemaker, implanted in a second operation up to 7 d later. Clinical details of the patients are available in Table 1; the first symptom for patients 8 and 12 were not available in the medical records. The mean percentage improvement in the motor section of the Unified Parkinson's Disease Rating Scale (UPDRS) following treatment with levodopa was 64.4 ± (SEM) 4.5% (p < 0.001, signed rank test) across subjects, indicating good responsiveness to levodopa in our study participants.

**Fig. 1:**
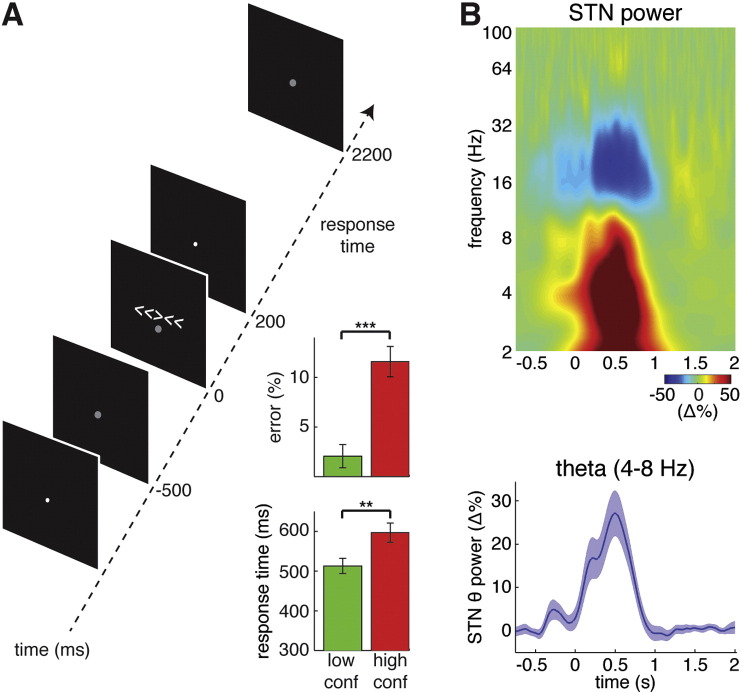
Task. (A) Patients performed an arrowed version of the Eriksen Flanker task, where they indicated the direction of a middle arrow flanker by either low (<<<<<) or high (>><>>) conflict arrows. A fixation dot was present throughout the entire experiment, but changed from white to gray 500 ms before the arrows were shown. Inset shows that subjects had slower reaction times and higher error rates during the high conflict trials. Group average ± SEM is shown. ** denotes p < 0.01, *** denotes p < 0.001. (B) Consistent with previous STN recordings, the onset of the arrows elicited a decrease in beta power and an increase in low frequency, including theta, power. Our subsequent analysis focused on the band passed theta (4–8 Hz) power (bottom). The group average across all trials and all STNs is shown for top and bottom panels, with shaded region in bottom panel denoting SEM across STNs.

Patients performed an arrow version of the flanker task (Eriksen and Eriksen, 1974) 3–6 d after electrode implantation. The task was identical to one we have previously used (Zavala et al., 2013), a schematic of which is shown in Fig. 1A. Each trial began with a black screen containing a white fixation dot in the middle of the screen, which subtended a visual angle of ≈ 1°. Five hundred milliseconds before the arrows were shown, the dot changed from white to gray to prepare the test subject for the imperative cue. Either congruent (> > > > >, henceforth referred to as “low conflict”) or incongruent (< < > < <, henceforth referred to as “high conflict”) arrows (visual angle ≈ 3° per arrow) were then briefly shown and replaced with the white fixation dot after 200 ms. The subjects had 2 more seconds in which to respond (2.2 s total possible window for a response) before the fixation dot changed from white to gray again to signal the next trial. Correct responses were indicated by a button press in the hand corresponding to the direction of the middle arrow. The ratio of high conflict trials to low conflict trials was 2:1, in order to increase the number of error trials collected, particularly as errors were infrequent. However, in the absence of a 1:1 ratio, it should be highlighted that trial types may have potentially differed in their saliency and priming. Subjects underwent two 60-trial blocks. Subjects were allowed to practice the task as long as they wished before the electrophysiological recordings were made. The practice sessions were generally quite short (< 20 trials) as the task was designed to be as simple as possible.

All patients performed the task while on their regular parkinsonian medication. In order to identify potential correlations between PD severity and behavior, reaction time during the task was correlated with baseline UPDRS scores that were recorded as part of the standard pre-surgical procedure. UPDRS scores on medication did not correlate with task performance as determined by accuracy rates (r = 0.03, p > 0.05, Spearman's correlation) or reaction times (r = − 0.43, p > 0.05, Spearman's correlation) across all trials. Off-medication UPDRS scores also did not correlate with performance (accuracy rate, r = − 0.21, p > 0.05; reaction time r = − 0.30, p > 0.05, Spearman's correlation). Two of the subjects showed significantly higher error rates than the other 11 subjects (error rate across all trials = 27.7% and 27.8% for the two outlier subjects and 8.5 ± 1.1% for the 11 other subjects) and were therefore excluded prior to any of the analysis. One of these two subjects had an impulse control disorder, and the other subject had reaction times that were slower then those recorded for the remaining subjects (1008 ms mean reaction time for excluded subject vs. 482–712 ms for the remaining subjects). Further justification for excluding these two subjects stems from their reaction time distributions, which showed no significant difference between low and high conflict trials (p > 0.05, rank sum-test). All other subjects did exhibit a significant within subject difference. In one of the retained subjects, recordings were only accessible from one STN, therefore the total number of STNs included in the analysis was 21. Data from two of the participants (cases 1 and 2) were also included in our previously published work (Zavala et al., 2013), although that work did not include mesial frontal theta connectivity, which is the main focus of this paper.

### LFP data recording and behavioral analysis

STN LFPs were recorded from the DBS electrodes. Simultaneously, continuous scalp EEG was recorded from frontal, central and parietal electrodes over the midline (Fz, Cz and Pz; International 10–20 System). More lateral electrodes were prohibited by surgical wounds and dressings in this patient group. All signals were sampled at 2048 Hz, bandpass filtered between 0.5 and 500 Hz, and amplified using a TMSi Porti and its respective software (TMS International). Monopolar recordings were subsequently converted off-line to a bipolar montage between adjacent contacts (three bipolar channels per STN side and two bipolar channels for the EEG recordings: Fz–Cz and Pz–Cz) to limit the effects of volume conduction from distant sources. The Fz–Cz electrode is thought to reflect activity from several structures of the medial frontal cortex implicated in theta rhythm generation such as the anterior cingulate cortex (Gevins et al., 1997, Pizzagalli et al., 2003, Wang et al., 2005). It was our hope that the Pz–Cz electrode could serve as a control site by recording the activity of the parietal cortex. Henceforth we will refer to these two bipolar contacts as mesial frontal and parietal cortex, respectively, although it should be kept in mind that both signals had the Cz electrode in common.

Data were analyzed using custom-written Matlab (MathWorks) scripts. Before further analysis, LFP data were filtered between 1 and 500 Hz and down-sampled to 1000 Hz. Any trials with reaction times > 1.5 s (including no response trials) or < 300 ms were not included in the analysis (< 1%). For the comparison of correct low conflict and high conflict trials, all incorrect responses were excluded. For the comparison of high conflict error trials to high conflict correct trials, a reaction time matched subset of the correct trials was used (Cavanagh et al., 2014). For each incorrect high conflict trial, one reaction time matched trial was randomly chosen from all of the correct high conflict trials that had a reaction time within 30 ms of the incorrect trial. Any incorrect trial that did not have a correct trial that was within 30 ms was excluded from the analysis. Subjects with fewer than 5 errors were not included in this analysis (n = 2). The average number of error trials for the 9 subjects that were included in the error analysis was 8 ± 0.8 trials.

For the analysis shown in Fig. 3, the correct trials that followed error trials were compared to the correct trials that followed the reaction time matched set of correct trials. The post-error trials and the post-reaction time matched correct trials had a similar proportion of high conflict trials that was consistent with the 2:1 ratio of high to low conflict trials that was used in the task (63.5 ± 8.1% for the post-error trials and 64.3 ± 4.3% for the post-reaction time matched correct trials, p = 0.87, signed rank test). Note that, although error trials were infrequent, they were evenly distributed across the recording blocks. The average number of trials used for this analysis was 6 ± 0.9 trials for the post-error trials and 6.1 ± 0.8 for the post-reaction time matched correct trials. These numbers were slightly lower than the number of error trials and reaction time matched correct trials because a few subjects had occasional back-to-back errors or an error that occurred at the end of a block. The low number of error trials used for this analysis is a significant shortcoming of both this study and the only previous study that has analyzed STN activity during across trial adaptations to errors (Cavanagh et al., 2014). In order to determine whether or not such a low number of trials could be reliably used to measure connectivity between the STN and the mesial frontal cortex, the comparison between low and high conflict correct trials was repeated using only 6 trials from each of the two categories (see the Methods section “Intersite phase coherence”, below).

### Analysis of theta power

The instantaneous theta power and phase of the bipolar LFP and EEG signals were calculated by bandpass filtering each trial's raw signal between 4 and 8 Hz and applying the Hilbert transform. Each trial was analyzed from 0.75 s before to 2 s after arrow onset for the cue-aligned analysis, from 1.5 s before to 1.5 s after the response for the response-aligned analysis, and from 2 s before to 0 s before arrow onset for the analysis of the pre-stimulus period (Fig. 4). A 1 s buffer on either side was used when calculating phase and power to eliminate any edge effects. Any trial with a clear artifact in any of the LFP or EEG channels was discarded.

To assess differences in power between low and high conflict trials, the following approach was used. First, the mean power in each bipolar recording for each trial type was calculated by averaging the power time series across trials. This method produced a time series for low and high conflict trials for each of the three bipolar contacts on each STN electrode and the two bipolar EEG contacts. Each time series was then normalized by determining the percentage change in power relative to the mean power of that channel during a “baseline” period. The baseline period consisted of the full second leading up to the warning cue onset before each trial (t = − 1.5 to − 0.5, relative to the arrow onset). Finally, all three STN bipolar contacts were averaged together for each side before being averaged across all STN electrodes. Averaging across all the contact pairs in a given electrode was performed so as to avoid selection bias, and support for this strategy comes from that fact that our previous studies have not allowed us to asses regional difference in STN reactivity based on LFP biomarkers (Zavala et al., 2013, Zavala et al., 2014).

To assess the statistical significance of any difference between low and high conflict trials, the across electrode average was repeated 1000 times with the low and high conflict labels of each electrode's average data randomly assigned during each permutation. The p value of each time point was found by comparing the actual mean difference to the distribution of the 1000 permutations. The p values were then corrected for multiple comparisons using exceedence mass testing (Maris and Oostenveld, 2007). Exceedence mass testing involves integrating the excess mass of suprathreshold clusters in the spectrogram and recording the largest per iteration. The top 5% of this distribution (p < 0.05) then determined the corrected threshold for time series-wise significance. When performing other comparisons (i.e., high conflict errors vs. high conflict correct, post-high conflict errors vs. post-high conflict correct, fast-high conflict vs. slow-high conflict), the same procedure was repeated using the relevant trial groups. Throughout all of our analyses, exceedence mass testing was used to correct for multiple comparisons whenever the difference in a continuous time series between two conditions was assessed. Where appropriate, ANOVAs were also used to test for any effects of STN side (right vs. left) and to confirm the results of the permutation test by averaging across a wider time window of 500 ms. A Kolmogorov–Smirnov test was used to confirm that the data included in the ANOVAs was normally distributed. For the laterality analysis, the one subject with STN recordings in only one hemisphere was excluded.

To assess for potential correlations between pre-stimulus theta power and reaction time, the correct high and low conflict trial groups were each median split into two groups consisting of the fastest and slowest half of trials in each trial type (Fig. 4). This division, together with the 2:1 ratio of high to low conflict trials used in the task resulted in an average of 18.1 ± 0.8 trials in each condition for the median split low conflict trials and 34.6 ± 2.0 trials in the median split high conflict trials. The results from this analysis were subsequently corroborated by analyzing the within-subject, single-trial correlation between reaction time and normalized power changes. To this end, the normalized theta power of each trial was averaged across the 1.25 s time period leading up to the warning cue onset and correlated with the reaction time in that trial. The resulting correlation coefficients (positive and negative coefficients derived using Spearman's correlation) were then Fisher transformed and averaged across the STN sides. A two-tailed, one-sample sign test was performed to determine whether the mean correlation was significantly different from zero across subjects.

In order to plot the average spectrogram across all correct trials (Fig. 1b), the power analysis was repeated using the Morlet wavelet at 8 scales/octave from 2 to 107 Hz. The data were then normalized and averaged across all STNs using the same procedure described above for the Hilbert theta power. The average beta power observed across all trials was subsequently used as an estimate of the bipolar contact pair closest to the dorsolateral motor territory of the STN (Brown, 2013).

### Intertrial phase consistency (ITPC)

To analyze the theta phase consistency across trials, the inter trial phase consistency (ITPC, sometimes also called inter trial phase clustering; Cohen and Gulbinaite, 2014) was found in at each time point by projecting the phase at time t for each trial onto the complex plane, averaging across trials, and taking the absolute value. Using this formulation, an ITPC(t) value of 0 would mean there is a uniform distribution of phase across trials at time t, and a value of 1 would mean that the phase at time t is identical for each trial. ITPC values were calculated separately for low and high conflict trials. In order to prevent the 2:1 high to low conflict trial ratio from affecting our results, the high conflict trials were down-sampled for each subject to match the number of low conflict trials. 1000 down-sampled ITPC time series were calculated for each subject's high conflict trials, and the average of the down-sampled values was used for each subject. The low conflict and the down-sampled high conflict ITPC time series were then normalized by determining the percentage change in ITPC relative to the mean “baseline” ITPC value recorded during the full second leading up to the presentation of the warning cue onset (t = − 1.5 to − 0.5, relative to the arrow onset). Randomly drawn subsets of all the trails were used to calculate the baseline 1000 times, with the number of trials used for each baseline calculation equal to the number of low conflict trials (and the number of down-sampled high conflict trials). An identical procedure was used when comparing error trials to correct trials, with the only exception being that the number of trials used to calculate the baseline was equal to the number of error trials.

To assess the statistical differences between conditions, the low and high conflict normalized ITPC values were first calculated for each bipolar signal and averaged across all three bipolar contact pairs of each STN electrode. The resulting values were then averaged across electrodes and the difference between the two trial types was compared to 1000 permuted differences generating by permuting each electrode's average values prior to finding the across-electrode average. The p value at each point was calculated using the distribution of the 1000 permuted values and corrected for multiple comparisons at a significance level of 0.05 using exceedence mass testing.

### Intersite phase coherence

The cortico:STN phase coherence (occasionally also referred to as inter-site phase clustering; Cohen and Gulbinaite, 2014) was calculated using the continuous time evolving methods we have previously used (Zavala et al., 2014) as outlined by Lachaux et al. (2002). Low- and high-conflict trials were analyzed separately, with the high conflict trial's phase coherence values being calculated 1000 times using a down-sampled data set (see ITPC section above). The difference between the instantaneous theta phase (projected on the complex plane) at time t in each bipolar STN contact and the theta phase at time t in the mesial frontal channel was found at each time point. The phase difference values at time t were then averaged across trials and a sliding window was used to integrate across time (Lachaux et al., 2002). The width of the window was chosen to be 333 ms (2 cycles of a 6 Hz oscillation). The magnitude of the resulting average was then taken to generate the phase coherence. Each channel's time evolving phase coherence signal was then normalized by that channel's “baseline” phase coherence. The baseline was chosen in the same way as it was chosen for the ITPC analysis: calculating the mean phase coherence value (averaged across the full second before the warning cue onset) 1000 times for down-sampled sets of trials (equal in number to the number of low conflict trials) randomly chosen from all of the trials. The three resulting normalized time series generated for each of the three contacts in each STN were then averaged within each STN before averaging across STNs. Statistical significance was determined using permutation testing as outlined above. To calculate the Hilbert parietal cortex:STN phase coherence, the same analysis was done using the Pz–Cz bipolar electrode instead of the Fz–Cz electrode. An identical procedure was used when comparing error trials to correct trials or when comparing post-error trials to post-reaction time matched correct trials. The only exception being that the number of trials used to calculate the baseline was equal to the number of trials used in the comparison.

Finally, in order to determine whether the low trial count used in the comparison of post-error trials and post-reaction time matched correct trials could be used to reliably estimate intersite phase coherence, we repeated the analysis of the correct high and low conflict trials using only 6 trials for each condition for each subject (Supplementary Fig. 1). This analysis was identical to that used when analyzing the whole dataset with the exception that rather then down-sample the high conflict trials to match the number of low conflict trials 1000 times, both trial types were down-sampled to 6 trials. Similarly, the baseline was down-sampled to 6 trials. On average, using only 6 trials to estimate the intersite phase coherence generated qualitatively similar differences between low and high conflict trials as those seen when the full data set was used. As would be expected, however, there were variations in the response patterns observed on individual iterations, which is why 1000 iterations were conducted. When comparing the post-error trials to the post-reaction time matched correct trials, we did not have the luxury of using 1000 iterations.

## Results

### Behavioral effects

Subjects performed an arrowed version of the Eriksen flanker task (Fig. 1a). Patients were significantly slower during the high conflict condition relative to the low conflict condition (mean ± SEM = 596 ± 24 ms vs. 512 ± 19 ms, p < 0.001, signed rank test) and showed a significantly higher error rate during conflict (mean ± SEM = 11.6 ± 1.5% vs. 2.1 ± 1.2%, p < 0.01, signed rank test; Fig. 1a, inset). All data reported in this manuscript are reported as mean ± SEM. There was no slowing of reaction time in trials that followed an error (p > 0.05, signed rank test). There was also no Gratton effect on reaction time (Gratton et al., 1992): the reaction time of high conflict trials was not affected by the level of conflict in the previous trial (p > 0.05, signed rank test ), and the same was true for low conflict trials (p > 0.05, signed rank test).

### Conflict related difference in STN LFP and mesial frontal theta activity

Subjects demonstrated an increase in theta power in the STN LFP during the task, as well as a decrease in beta power (top panel Fig. 1b). As most of the literature concerning cortical and subcortical conflict related networks implicates coupling in the theta band (Cavanagh et al., 2011, Cavanagh et al., 2012, Zavala et al., 2013, Zavala et al., 2014, Zavala et al., 2015a), we focused our attention on this band. The bottom panel of Fig. 1b shows that during the task, the subjects showed a consistent increase in band-passed theta (4 to 8 Hz) LFP power in the STN LFP after onset of the warning cue (at t = − 500 ms), and this was followed by an even greater increase after onset of the arrows (at t = 0 ms).

**Fig. 2:**
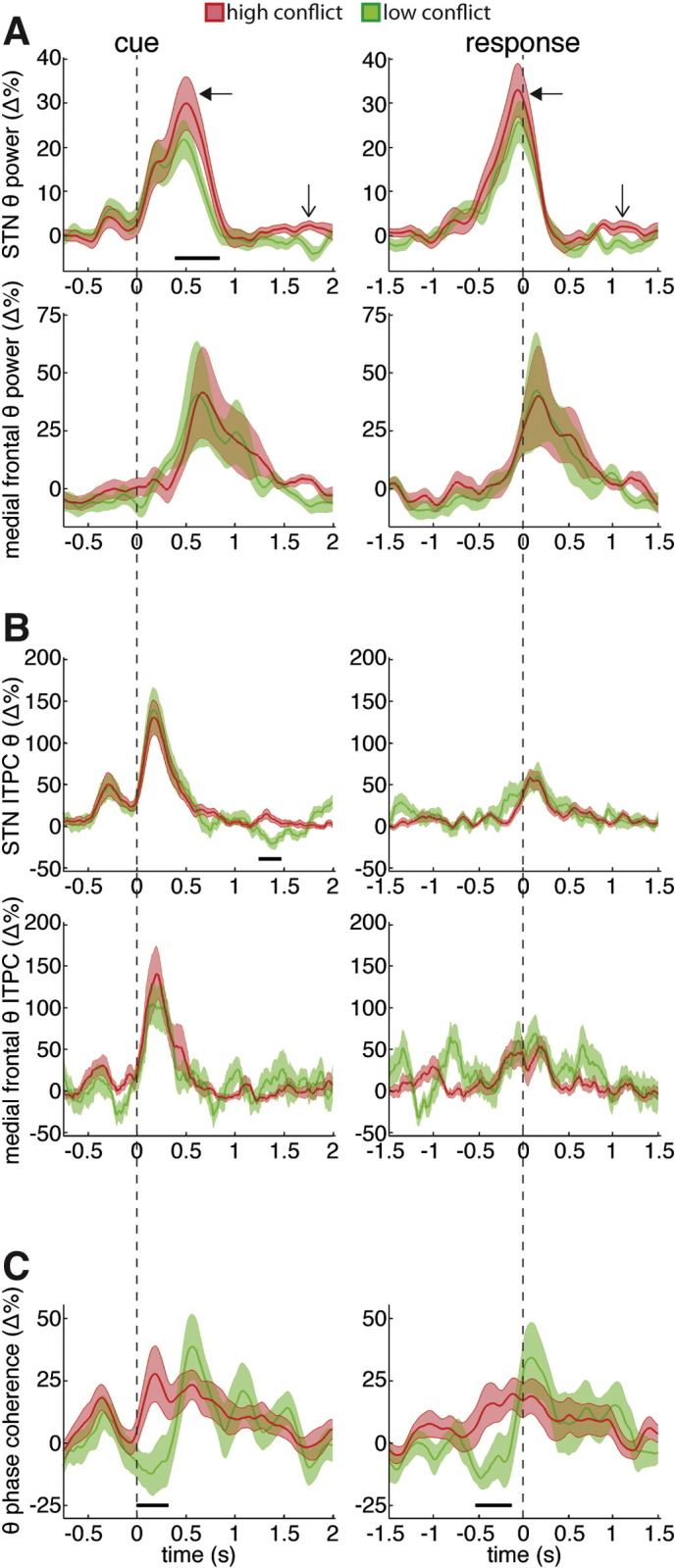
Conflict related theta activity in the STN and mesial frontal cortex. (A) Top row shows STN theta power aligned such that t = 0 corresponds to the stimulus onset (left) or the response (right). High conflict trials showed significantly higher pre-response STN theta power (see solid arrows and black bars). A post-response difference can also be seen as the post-high conflict theta power is higher during the “baseline” period of the subsequent trial (see line arrows). Black line denotes significant time points that survived correction for multiple comparisons (p < 0.05, permutation testing). Group average percentage change ± SEM are shown for all plots. Bottom row is same as top row but for mesial frontal theta power. Note STN theta power peaked before the response while mesial frontal theta power peaked after the response. (B) Same as A, but for inter-trial phase consistency (ITPC). There were no significant time points that showed a conflict related difference. Note both the STN and mesial frontal cortex increases in ITPC occurred at the same time and earlier than the theta power increases in either site. (C) Same as A, but for the inter-site phase coherence. High conflict trials showed significantly higher pre-response coherence between the STN and the mesial frontal. This increase occurred at the same time as the increase in ITPC, suggesting that though both low and high conflict trials show a increase in phase consistency early in the trial (before the theta power increase), only during conflict are the phases synchronized between the two brain regions.

In order to explore the temporal evolution of theta band activity during the flanker task, we analyzed the theta power, theta inter-trial phase consistency (ITPC), and theta inter-site phase coherence in the STN LFP and in the mesial frontal EEG (Fig. 2). High conflict trials were associated with a higher increase in pre-response theta power in the STN LFP relative to the low conflict trials (Fig. 2a, top). During the 500 ms leading up to the response, the average theta power increase was 15.7 ± 3.7% for low conflict trials and 21.4 ± 4.6% for high conflict trials. These differences were significant and a two way ANOVA did not reveal a dependence on laterality (ANOVA, within-subject repeated measures, conflict × STN side (left vs. right): conflict F = 5.57, p < 0.05; STN side F = 0.05, p = 0.83; interaction F = 1.44, p = 0.26). When we analyzed the mesial frontal theta power changes (Fig. 2a, bottom), we observed no significant conflict related differences. In line with previous scalp EEG studies (Cavanagh et al., 2009, Cavanagh et al., 2012, Cohen and van Gaal, 2014), the mesial frontal theta activity peaked after the response (mean peak time ± SEM for all correct trials = 85.9 ± 78.6 ms relative to the response), which was significantly later than the STN theta power peak that occurred before the response (mean peak time ± SEM for all correct trials = − 58.4 ± 28.1 ms relative to the response, p < 0.05, paired t-test; relative to the cue these values were 770 ± 82.5 ms for the mesial frontal EEG and 633.7 ± 94.1 ms for the STN LFP, p < 0.05, paired t-test).

Following the presentation of the warning cue and of the target arrows, STN LFP mesial frontal EEG theta oscillations demonstrated theta phase locking to the stimulus onset as indexed by ITPC. Both STN LFP and mesial frontal EEG ITPC increases were much higher for the cue aligned data than they were for the response aligned data. Notably, the STN LFP and mesial frontal EEG ITPC increases seemed to occur simultaneously, early during the trial (mean STN LFP ITPC peak time ± SEM for all correct trials = 257.6 ± 49.8 ms relative to the cue; peak time for medial frontal EEG ITPC = 319.5 ± 115.6 ms, p > 0.6, paired t-test), and they both peaked significantly (p < 0.001, paired t-test) before the theta power increases described above. Though there were no significant, conflict related differences in the stimulus-triggered ITPC increase in either location, this may be because it is not the particular phase of the oscillation that is important at any given time in a trial, but rather the coherence of the phases between the STN LFP and mesial frontal EEG.

Consistent with the idea that coherent oscillations between two brain sites would allow behaviorally relevant information to flow from one site to the other (Fries, 2005), we observed higher pre-response theta phase coherence between the mesial frontal EEG and the STN LFP during the high conflict trials (Fig. 2c). Averaging across the first 500 ms following the cue showed a significant increase (relative to baseline) in phase coherence for the high conflict trials (20.0 ± 7.1%, p < 0.05, one-sample t-test) and an unchanged value for the low conflict trials (− 2.4 ± 7.8%, p = 0.8, one-sample t-test). The differences between low and high conflict trials were significant and a two way ANOVA did not reveal a dependence on laterality (ANOVA, within-subject repeated measures, conflict × STN side (left vs. right): conflict F = 9.32, p < 0.05; STN side F = 1.36, p = 0.27; interaction F = 1.51, p = 0.25). Likewise looking at the 500 ms leading up to the response showed a significant increase in phase coherence for the high conflict condition (16.4 ± 4.6%, p < 0.01, one-sample t-test) and no change in the low conflict condition (− 3.1 ± 7.0%, p = 0.5, one-sample t-test). Once again these differences were significant and a two way ANOVA did not reveal a dependence on laterality (ANOVA, within-subject repeated measures, conflict × STN side (left vs. right): conflict F = 12.77, p < 0.01; STN side F = 2.36, p = 0.16; interaction F = 1.27, p = 0.29). It is important to note that changes in intersite phase coherence can occur without changes in ITPC (Zavala et al., 2014, Zavala et al., 2016), which is what is occurring in the 500 ms leading up to the response. As a control, we also analyzed the theta band phase coherence between the STN LFP and EEG over the parietal cortex (Pz–Cz). This revealed no conflict related differences in the continuous time series data (Supplementary Fig. 2) or when the phase coherence was averaged across the first 500 ms following the cue (ANOVA, within-subject repeated measures, conflict × STN side (left vs. right): conflict F = 1.82, p = 0.21; STN side F = 1.05, p = 0.33; interaction F = 0.13, p = 0.73).

We found no systematic distribution of theta band cortico:STN phase coherence across STN electrodes. To this end we compared theta cortico:STN phase coherence in the dorsal-most contact bipolar pair of each STN electrode to the cortico:STN phase coherence in the ventral-most contact bipolar pair of each STN electrode during the first 500 ms following the stimulus. This revealed that the higher theta phase coherence observed during high-conflict trials was independent of STN electrode location (ANOVA, within-subject repeated-measures, conflict × channel: conflict, F = 7.74, df = 1, p = 0.0115; channel, F = 3.27, df = 1, p = 0.09; interaction, F = 0.03, df = 1 p = 0.58). Similar results were obtained when we repeated this analysis using the assumption that the bipolar contact pair with the highest beta band power in each electrode was in or nearest to the dorsolateral motor territory of the STN (for review, see Brown, 2013). Nineteen of the STNs contained at least one bipolar contact that was ventral to the contact with the highest beta power. Comparing the changes in theta band phase coherence observed over the highest beta contact with the changes in phase coherence observed over the remaining ventral contacts revealed that the higher theta phase coherence observed during the high conflict trials was independent of beta power localization (ANOVA, within-subject repeated-measures, conflict × channel: conflict, F = 13.93, df = 1, p = 0.002; channel, F = 1.56, df = 1, p = 0.23; interaction, F = 0.07, df = 1 p = 0.79).

The conflict related increase in phase coherence was not secondary to ITPC differences, as neither the STN LFP nor mesial frontal EEG showed any significant differences in ITPC for low or high conflict trials over these periods. Furthermore, we observed no correlation across subjects between the average STN ITPC and the average STN-frontal EEG theta phase coherence during the first 500 ms following the cue (r = 0.27, p > 0.05 for high conflict trials and r = 0.20, p > 0.05 for low conflict trials, Spearman's Correlation). However, given that the stimulus onset induced a phase reset in both the STN LFP and the mesial frontal EEG for low conflict trials, it is interesting that the low conflict trials did not show any increases in phase coherence during the periods with elevated ITPC levels. This discrepancy, together with the fact that the phase consistency across trials is not uniform (average maximum un-normalized ITPC across all correct trials in all subjects = 0.34 ± 0.03, perfect inter trial phase alignments would have a value of 1) shows that there is some inconsistency in the exact phase to which both structures are aligning. Only during conflict did the two structures reset their phase in a way such that phase differences were sustained across time both within and across trials (see also Nigbur et al., 2012). The sustained consistency in phase difference likely explains why the response aligned data also showed a conflict related difference in phase coherence, despite the fact that the ITPC increases were locked to the cue and not the response.

### Post-error related difference in STN LFP and mesial frontal EEG theta phase coherence

**Fig. 3:**
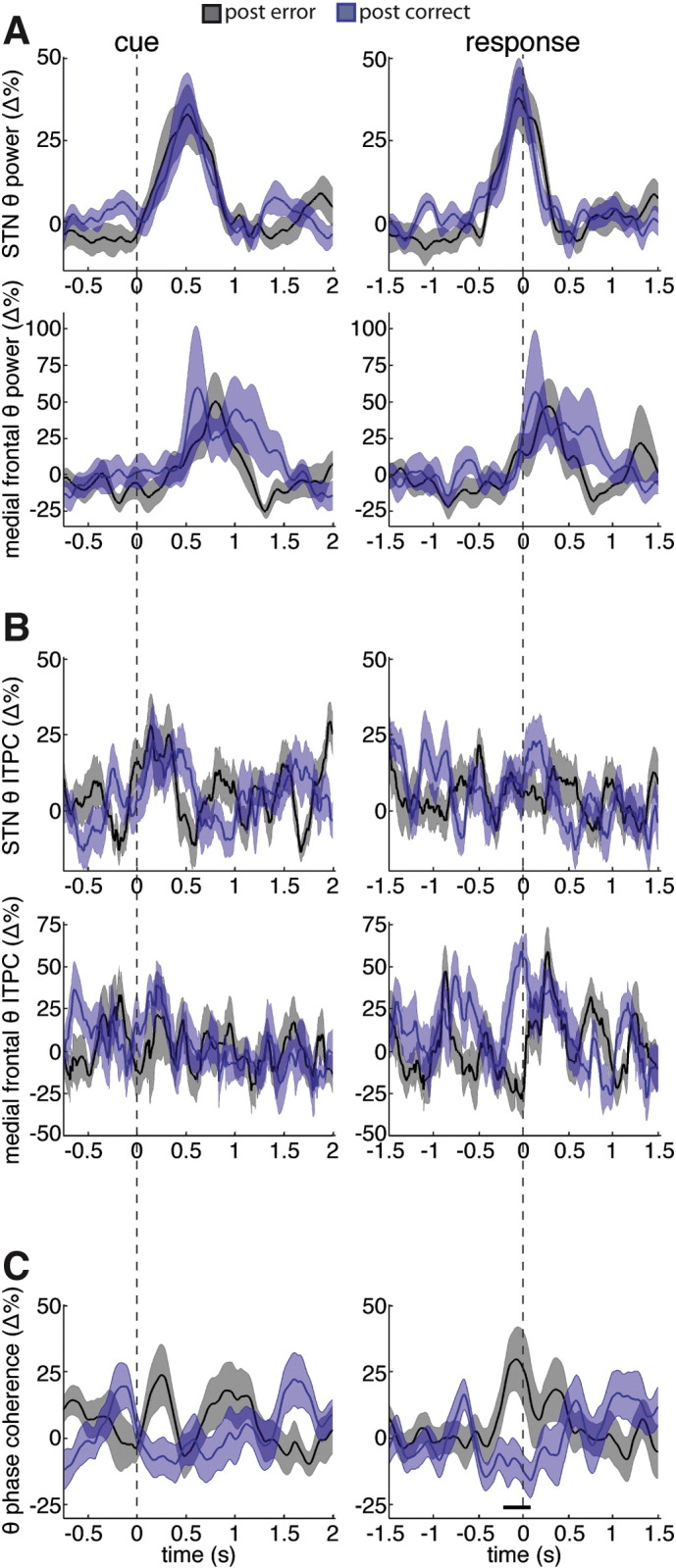
Theta coherence between STN and mesial frontal cortex is higher on trials that followed an error. Same as Fig. 2 (panel A is theta power panel B is theta ITPC, and panel C is theta phase coherence), but for the correct trials (black trace) that took place when an error was committed on the previous trial (not shown). Correct trials that followed a correct trial that was reaction time matched with an error trial are shown in blue. Black line denotes significant time points that survived correction for multiple comparisons (p < 0.05, permutation testing).

Much of the literature concerning the mPFC focuses on the role this brain area might play in error monitoring and post-error adaptations (Ridderinkhof et al., 2004, Danielmeier and Ullsperger, 2011). When we compared the theta band activity in the incorrect high conflict trials to the activity observed in a reaction time matched set of correct high conflict trials, we observed no differences in theta band power, ITPC, or phase coherence (data not shown). We also observed no differences in theta power or ITPC when we compared the correct trial that followed a high conflict incorrect trial to the correct trial that followed a reaction time matched high conflict correct trial (Fig. 3a,b). However, when we analyzed the phase coherence between the mesial frontal EEG and the STN LFP, we observed a significant, pre-response difference between the trials that followed errors and those that did not (Fig. 3c). Averaging across the 500 ms leading up to the response revealed significantly higher phase coherence in the trials that followed an error (post-error coherence = 13.3 ± 6.8%, post-correct coherence = − 9.9 ± 4.4%, ANOVA, within-subject repeated measures, accuracy (post-error trials vs. post-correct trials) × STN side (left vs. right): accuracy F = 6.07, p < 0.05; STN side F = 3.58, p = 0.10; interaction F = 0.78, p = 0.41). It is important to note, however, that the increase in phase coherence observed during the trials that followed an error was not significantly different from zero (p = 0.07, one-sample t-test). As a control, we also analyzed the theta band phase coherence between the STN LFP and the EEG over the parietal cortex (Pz–Cz) and found no error related differences (data not shown).

### Pre-stimulus correlates of conflict and behavior

**Fig. 4:**
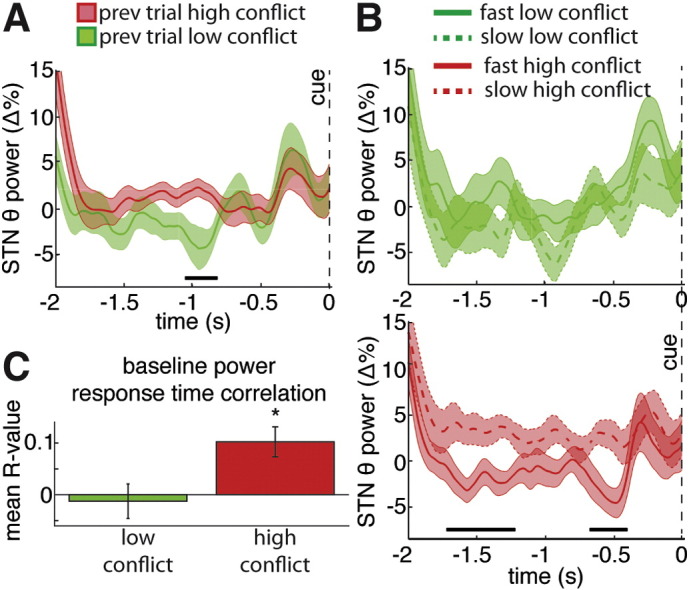
Inter-trial theta power is related to slower reaction times during conflict. (A) The theta power (group average ± SEM) during the time periods that followed low and high conflict trials is shown aligned to the onset of the arrows for the subsequent trial (t = 0). High conflict trials were followed by significantly higher theta power during the “baseline” of the next trial. Black line denotes significant time points that survived correction for multiple comparisons (p < 0.05, permutation testing). (B) A median split of the low and high conflict trials based on reaction time revealed that the slowest high conflict had higher pre-warning cue theta power than the fastest high conflict trials. (C) High conflict trial reaction time significantly correlated with the theta power that preceded the warning cue onset (t = − 1.75 to −.5, relative to arrow onset at t = 0). This effect was not present during low conflict trials. Across-subject averages of within-subject Spearman correlation coefficients (Fisher transformed) are shown for each condition (average ± SEM). * denotes significantly non-zero correlation coefficient (p < 0.05).

As the previous section highlighted that task related activity on one trial can be related to electrophysiological activity on the subsequent trial, we decided to further explore the post-response, pre-subsequent warning cue STN power differences reported in Fig. 2a. When we analyzed the pre-stimulus periods (t = − 2000 to 0 ms) that followed high and low conflict trials, we observed a significant difference in power approximately midway through this period (Fig. 4a). This difference did not reflect a “spill over” of the conflict related differences of the previous trial as there was a period in between the pre-response differences of the previous trial and the pre-stimulus differences on the subsequent trial in which the power had returned to baseline levels for both conditions. Moreover, when we median split high conflict trials into two populations based on whether they were in the fastest half or the slowest half of the high conflict trials, we observed significant differences in the pre-warning cue power levels between the fastest and slowest high conflict trials (Fig. 4b, bottom). During the 1.25 s that preceded the warning cue, the power level for the slowest high conflict trials was 2.8 ± 0.7% higher than the average baseline power observed during all trials, and the power level for the fastest high conflict trials was 1.7 ± 0.6% lower than the average baseline (ANOVA, within-subject repeated measures, speed (fastest high conflict trials vs. slowest high conflict trials) × STN side (left vs. right): accuracy F = 7.7, p < 0.05; STN side F = 0.09, p = 0.77; interaction F = 3.35, p = 0.10). This effect was not present during the low conflict trials (Fig. 4b, top), suggesting that the pre-stimulus differences only affect reaction time when the subsequent stimulus contains conflict. Further support for this claim stems from our finding that the pre-stimulus power levels significantly correlated with trial reaction time in the high conflict condition (mean Fisher transformed R = 0.10 ± 0.03, p < 0.05, one-sample sign test), but not in the low conflict condition (mean Fisher transformed R = − 0.01 ± 0.03, p = 0.9, one-sample sign test, Fig. 4c).

## Discussion

### Study summary and limitations

The results we present here corroborate previous findings concerning the potentially crucial roles of theta oscillations and of the STN during conflict (Cavanagh et al., 2011, Zavala et al., 2013, Zavala et al., 2014). Our results also provide several novel insights concerning the relationships between the STN and cognitive control. High conflict trials demonstrated higher theta phase coupling between the mesial frontal EEG and the STN, and these differences occurred early in the trial before any power differences took place. Furthermore, error trials were followed by increased phase coherence between the two sites on the subsequent trial. Finally, pre-stimulus differences in STN theta power correlated with the reaction time of the subsequent trial, but only when that trial contained conflict. These data help explain why disruption of this network by DBS may influence behaviors such as impulsivity during conflict and suggest the hitherto untested hypothesis that DBS may also interfere with some of the behavioral adjustments that take place when mistakes are committed.

Prior to further discussing the significance of our results, it is important to discuss some of the limitations in this study. First and foremost, this study was, out of necessity, conducted in patients with Parkinson's disease. Nevertheless, the patients performed the task on their regular medication in an attempt to reproduce “normal” basal ganglia activity to the greatest extent possible, although Parkinson's disease medications have been shown to alter theta band activity in a patients with impulse control disorders (Rodriguez-Oroz et al., 2011). Further evidence supporting the generalizability of our findings stems from the fact that the subjects included in the analysis showed relatively fast reaction times (about 500 ms on average for low conflict trials), that conflict slowed reaction times as in healthy subjects, and that behavioral performance did not correlate with UPDRS scores across subjects. The second limitation of this study is the low number of errors that we were able to record per subject. Though we were able to show that the differences between the low and high conflict trials could be reproduced using only 6 trials in each condition per subject, the post-error trial results should still be interpreted with caution and corroborated with future studies using a task designed to explicitly explore across trial adaptations. In line with this, the low trial counts may be the reason why we were unable to reproduce previous conflict or error related differences in mPFC theta power (Cavanagh et al., 2012, Cohen and van Gaal, 2014). However, ours is not the only study that has failed to show these differences when averaging across trials (Cohen and Cavanagh, 2011). The third major limitation of the present study was the limited EEG spatial sampling, which prohibited any source-based analysis of the EEG. This limitation was dictated by the need to avoid surgical sites and dressings.

### Conflict related STN theta power and phase play separate roles within a trial

Despite the above caveats, we believe our data allow us to make some inferences regarding theta band interactions between the mPFC and the STN. The importance of mPFC–STN theta connectivity was previously suggested by a study showing that DBS to the STN disrupts the relationships between mPFC theta power and conflict related changes in reaction time (Cavanagh et al., 2011). To our knowledge, however, only one study has directly shown increased coherence between the mPFC and the STN during conflict (Zavala et al., 2014). The latter study used a gradually adapting dot motion task to show that trials containing slow increases in conflicting information were associated with increased mPFC–STN coherence and of mPFC drive of STN theta activity. Here, we have reproduced the increase in mPFC–STN phase coherence using the flanker task, which involves rapid onset conflict and ITPC increases. In contrast to the phase coherence, though, we did not observe any conflict related differences in ITPC during the task. This suggests that only when phases entrain in a way that produces a specific and sustained phase difference between the two structures does activity in the two structures become coherent (Fries, 2005, Nigbur et al., 2012). The finding may also help explain previous contradictory results between studies showing task related differences in ITPC in the mPFC (Cavanagh et al., 2012) and STN (Zavala et al., 2013), and others that do not (Cohen and Cavanagh, 2011, Nigbur et al., 2012, Zavala et al., 2014). It is the relative phase difference between mPFC and STN activities that is important, not the absolute phase in each.

The rapid conflict onset paradigm we used here also allows us to make claims regarding the relative timing of theta activity. One unexpected finding in our data was that STN LFP theta power increases actually occurred before those of the mesial frontal EEG. Indeed, the mesial frontal theta increase peaked after the response. Given that we have previously shown that mesial frontal oscillations drive those in the STN during conflict, this finding would seem paradoxical. A potential resolution is provided by the phase of the theta oscillations. Unlike the theta power increases, both the mesial frontal EEG and the STN LFP ITPC increases occurred at the same time, early during the trial, and it was these time periods that were associated with increased mesial frontal-STN coherence. The late increases in mesial frontal and STN theta power may therefore reflect other activity unrelated to whether or not conflict is present. Indeed, recent studies have shown that increases in mesial frontal theta power are separate from theta phase realignments (Cohen and Donner, 2013) and associated with EMG detected “partial” errors (Cohen and van Gaal, 2014). Interestingly, theta power differences between correct trials and partial error trials did not take place until after the partial error began. In light of these findings, we propose that *early* (phase coherence) changes in mesial frontal-STN theta coupling may be responsible for delaying all responses only during conflict trials, while *late* (power) changes in theta band activity may be responsible for suppressing only the incorrect response during all trials. This hypothesis may help explain why low conflict trials also show a late increase in STN theta power as well as a late increase in mesial frontal-STN phase coherence. According to this interpretation, the higher increases in theta power that occur late during high conflict trials may reflect a greater drive needed to inhibit the incorrect response due to a stronger activation of that response by the flanking arrows. Still, it must be stressed that the evidence we have presented is correlative in nature, and causality remain to be established.

### STN theta activity also plays a role across trials

Another key finding we report is that mesial frontal-STN connectivity seems to be involved in post-error monitoring, which is thought to be one of the core mPFC functions (Ridderinkhof et al., 2004, Danielmeier and Ullsperger, 2011). Error trials are associated with increased mPFC BOLD activity and theta power (Carter et al., 1998, Kerns et al., 2004, Cavanagh et al., 2009, Cavanagh et al., 2012). Furthermore, mPFC theta power is thought to underlie post-error slowing (Rabbitt, 1966), either by interactions with DLPFC (Kerns et al., 2004, Hanslmayr et al., 2007, Cavanagh et al., 2009, Cohen and Cavanagh, 2011, Cohen and van Gaal, 2014) or by adjusting the excitability of the motor cortex following errors (Danielmeier et al., 2011). Here, we show that the STN provides a path by which mPFC theta oscillations might influence the excitability of the motor cortex following errors and thus potentially allow for error-related behavioral adjustments. Nevertheless, in our paradigm there was no slowing of reaction time in trials that followed an error, perhaps because of our use of an inter trial interval that is too long for post-error slowing in healthy subjects (Jentzsch and Dudschig, 2009, Danielmeier and Ullsperger, 2011) or possibly because mesial frontal-STN connectivity might be impaired in our patient group, either as a function of PD or due to temporary stun effects at the level of the STN following surgery (Mann et al., 2009).

Finally, we observed that high conflict trials were followed by elevated STN LFP theta power levels in the baseline period before the subsequent trial, and that the reaction time in high conflict trials positively (albeit, weakly) correlated with that trial's baseline theta power. During the time periods showing significant differences in baseline power, there were no changes occurring on the screen that could have triggered a sensory response. In particular, the subject was yet to be given the warning cue to prepare for the next trial (this occurred at t = − 500 ms). Accordingly we posit that the difference in baseline power following high and low conflict trials may be related to the evaluation of the previous trial and a reconfiguration that may stem from this in anticipation of the next trial. In line with this, and against this feature being spurious and noise related, there was a correlation between the higher baseline power following high conflict trials and the reaction time in the subsequent trial. Interestingly, the baseline theta levels did not seem to influence low conflict trial reaction time, which is in line with studies showing that some theta activity only correlates with behavior during conflict (Cavanagh et al., 2011, Oehrn et al., 2014). Though our results are consistent with the posited braking effect of conflict related STN theta activity, they are in disagreement with what would be expected in the context of the Gratton effect (Gratton et al., 1992), which would predict *faster* responses during high conflict trials that follow a high conflict trials. That said, our paradigm elicited no Gratton effect on reaction time here or previously (Zavala et al., 2013), which may be related to impaired congruency sequence effects in PD (Rustamov et al., 2013). Taken together, the across trial effects we observed suggest that STN theta power is higher after high conflict trials and that higher pretrial theta power corresponds to slower reaction times during conflict. Whether this relationship only holds in the context of PD remains to be determined.

### Conclusion

We have investigated the mechanisms by which communication between brain regions may rapidly influence behavior. However, the picture presented is likely to be incomplete, as many studies have shown that mPFC–DLPFC interactions are also related to within trial conflict processing and across trial adjustments to conflict and errors (Kerns et al., 2004, Hanslmayr et al., 2007, Cavanagh et al., 2009, Nigbur et al., 2012, Cohen and van Gaal, 2014, Oehrn et al., 2014). Nevertheless, our study suggests that theta synchronization between cortical and subcortical structures may play a role in conflict and error related adaptations. Though disruption of this theta activity by either DBS (Cavanagh et al., 2011) or dopaminergic medication (Rodriguez-Oroz et al., 2011) has been shown to influence impulsivity, it remains to be determined if DBS affects across trial adjustments in behavior or whether other disorders involving impaired decision making and poor impulse control demonstrate altered theta activity in these networks (Fitzgerald et al., 2005, Van Meel et al., 2007).

## Figures and Tables

**Table 1:** Clinical details. UPDRS = Part III motor score of the United Parkinson's Disease Rating Scale. The first symptoms for patients 8 and 12 were not available.

Case	Age	Disease Duration	UPDRS Off (III)	UPDRS On (III)	First symptom	Reasons for surgery	Daily medication (mg/d)
1	58	10	42	20	Leg cramp	Tremor	Trihexyphenidyl 3 Levodopa 600 Rasagiline 1 Amantadine 100
2	62	10	20	8	Left side tremor & bradykinesia	On/off fluctuations, tremor & impulse control disorder	Levodopa 1000 Trihexyphenidyl 6
3	61	4	37	15	Left side tremor	Tremor	Amantadine 200 Levodopa 750 Entacapone 1000
4	65	15	51	21	Left hand tremor	Freezing	Amantadine 200 Levodopa 400 Ropinirole 12
5	44	10	33	7	Left hand tremor	Motor fluctuations, dyskinesia	Amantadine 200 Ropinirole 24 Rasagiline 1 Levodopa 600 Apomorphine 4.5 mg/h
6	42	9	60	42	Loss of dexterity	Bradykinesia, dystonia, freezing	Amantadine 400 Levodopa 600
7	51	9	56	12	Tremor	Motor fluctuations, dyskinesia	Apomorphine 4 Levodopa 1500 Entacapone 600
8	43	10	36	6		Motor fluctuations	Levodopa 600 Ropinirole 2
9	59	16	54	9	Loss of dexterity	Dyskinesia, painful cramps	Levodopa 100 Apomorphine 5 mg/h Rotigotine 4
10	69	10	34	12	Shuffling gait	Motor fluctuations, bradykinesia	Levodopa 800 Amantadine 100
11	53	7	25	5	Loss of dexterity	Dyskinesia, bradykinesia	Levodopa 800 Entacapone 800 Rasagiline 1
12	67	12	25	9		Off periods, & dyskinesia	Levodopa 950 Amantadine 100 Pramipexole 2.25
13	63	13	32	18	Dragging of left leg	Stiffness	Levodopa 700 Entacapone 1000 Ropinirole 8 Quetiapine 25 Clonazepam 0.5

## Appendix A Supplementary data

Supplementary figures. Image 1

## References

[bb0005] Bastin J., Polosan M., Benis D., Goetz L., Bhattacharjee M., Piallat B., Krainik A., Bougerol T., Chabardès S., David O. (2014). Inhibitory control and error monitoring by human subthalamic neurons. Transl. Psychiatry.

[bb0010] Botvinick M.M., Braver T.S., Barch D.M., Carter C.S., Cohen J.D. (2001). Conflict monitoring and cognitive control. Psychol. Rev..

[bb0015] Botvinick M.M., Cohen J.D., Carter C.S. (2004). Conflict monitoring and anterior cingulate cortex: an update. Trends Cogn. Sci..

[bb0020] Brittain J.-S., Watkins K.E., Joundi R.A., Ray N.J., Holland P., Green A.L., Aziz T.Z., Jenkinson N. (2012). A role for the subthalamic nucleus in response inhibition during conflict. J. Neurosci..

[bb0025] Brown P. (2013). Making use of pathological synchrony in Parkinson's disease. Clin. Neurophysiol..

[bb0030] Brown P., Chen C.C., Wang S., Kühn A.A., Doyle L., Yarrow K., Nuttin B., Stein J., Aziz T. (2006). Involvement of human basal ganglia in offline feedback control of voluntary movement. Curr. Biol. CB.

[bb0035] Carter C.S., Braver T.S., Barch D.M., Botvinick M.M., Noll D., Cohen J.D. (1998). Anterior cingulate cortex, error detection, and the online monitoring of performance. Science.

[bb0040] Cavanagh J.F., Cohen M.X., Allen J.J. (2009). Prelude to and resolution of an error: EEG phase synchrony reveals cognitive control dynamics during action monitoring. J. Neurosci..

[bb0045] Cavanagh J.F., Wiecki T.V., Cohen M.X., Figueroa C.M., Samanta J., Sherman S.J., Frank M.J. (2011). Subthalamic nucleus stimulation reverses mediofrontal influence over decision threshold. Nat. Neurosci..

[bb0050] Cavanagh J.F., Zambrano-Vazquez L., Allen J.J.B. (2012). Theta lingua franca: a common mid-frontal substrate for action monitoring processes. Psychophysiology.

[bb0055] Cavanagh J.F., Sanguinetti J.L., Allen J.J.B., Sherman S.J., Frank M.J. (2014). The subthalamic nucleus contributes to post-error slowing. J. Cogn. Neurosci..

[bb0060] Cohen M.X., Cavanagh J.F. (2011). Single-trial regression elucidates the role of prefrontal theta oscillations in response conflict. Front Percept. Sci..

[bb0065] Cohen M.X., Donner T.H. (2013). Midfrontal conflict-related theta-band power reflects neural oscillations that predict behavior. J. Neurophysiol.

[bb0070] Cohen M.X., Gulbinaite R. (2014). Five methodological challenges in cognitive electrophysiology. NeuroImage.

[bb0075] Cohen M.X., van Gaal S. (2014). Subthreshold muscle twitches dissociate oscillatory neural signatures of conflicts from errors. NeuroImage.

[bb0080] Coulthard E.J., Bogacz R., Javed S., Mooney L.K., Murphy G., Keeley S., Whone A.L. (2012). Distinct roles of dopamine and subthalamic nucleus in learning and probabilistic decision making. Brain.

[bb0085] Danielmeier C., Ullsperger M. (2011). Post-error adjustments. Front. Psychol..

[bb0090] Danielmeier C., Eichele T., Forstmann B.U., Tittgemeyer M., Ullsperger M. (2011). Posterior medial frontal cortex activity predicts post-error adaptations in task-related visual and motor areas. J. Neurosci..

[bb9000] Eriksen B.A., Eriksen C.W. (1974). Effects of noise letters upon the identification of a target letter in a nonsearch task. Perception & Psychophysics.

[bb0095] Fitzgerald K.D., Welsh R.C., Gehring W.J., Abelson J.L., Himle J.A., Liberzon I., Taylor S.F. (2005). Error-related hyperactivity of the anterior cingulate cortex in obsessive–compulsive disorder. Biol. Psychiatry.

[bb0100] Foltynie T., Hariz M.I. (2010). Surgical management of Parkinson's disease. Expert. Rev. Neurother..

[bb0105] Frank M.J., Samanta J., Moustafa A.A., Sherman S.J. (2007). Hold your horses: impulsivity, deep brain stimulation, and medication in parkinsonism. Science.

[bb0110] Fries P. (2005). A mechanism for cognitive dynamics: neuronal communication through neuronal coherence. Trends Cogn. Sci..

[bb0115] Fumagalli M., Giannicola G., Rosa M., Marceglia S., Lucchiari C., Mrakic-Sposta S., Servello D., Pacchetti C., Porta M., Sassi M., Zangaglia R., Franzini A., Albanese A., Romito L., Piacentini S., Zago S., Pravettoni G., Barbieri S., Priori A. (2011). Conflict-dependent dynamic of subthalamic nucleus oscillations during moral decisions. Soc. Neurosci..

[bb0120] Gevins A., Smith M.E., McEvoy L., Yu D. (1997). High-resolution EEG mapping of cortical activation related to working memory: effects of task difficulty, type of processing, and practice. Cereb Cortex New York N.

[bb0125] Gratton G., Coles M.G.H., Donchin E. (1992). Optimizing the use of information: strategic control of activation of responses. J. Exp. Psychol. Gen..

[bb0130] Green N., Bogacz R., Huebl J., Beyer A.-K., Kühn A.A., Heekeren H.R. (2013). Reduction of influence of task difficulty on perceptual decision making by STN deep brain stimulation. Curr. Biol..

[bb0135] Hanslmayr S., Pastötter B., Bäuml K.-H., Gruber S., Wimber M., Klimesch W. (2007). The electrophysiological dynamics of interference during the Stroop task. J. Cogn. Neurosci..

[bb0140] Jentzsch I., Dudschig C. (2009). Why do we slow down after an error? Mechanisms underlying the effects of posterror slowing. Q. J. Exp. Psychol..

[bb0145] Kerns J.G., Cohen J.D., MacDonald A.W., Cho R.Y., Stenger V.A., Carter C.S. (2004). Anterior cingulate conflict monitoring and adjustments in control. Science.

[bb0150] Lachaux J.-P., Lutz A., Rudrauf D., Cosmelli D., Le Van Quyen M., Martinerie J., Varela F. (2002). Estimating the time-course of coherence between single-trial brain signals: an introduction to wavelet coherence. Neurol. Clin. Neurophysiol..

[bb0155] Mann J.M., Foote K.D., Garvan C.W., Fernandez H.H., Jacobson C.E., Rodriguez R.L., Haq I.U., Siddiqui M.S., Malaty I.A., Morishita T., Hass C.J., Okun M.S. (2009). Brain penetration effects of microelectrodes and DBS leads in STN or GPi. J. Neurol. Neurosurg. Psychiatry.

[bb0160] Maris E., Oostenveld R. (2007). Nonparametric statistical testing of EEG- and MEG-data. J. Neurosci. Methods.

[bb0165] Nigbur R., Cohen M.X., Ridderinkhof K.R., Stürmer B. (2012). Theta dynamics reveal domain-specific control over stimulus and response conflict. J. Cogn. Neurosci..

[bb0170] Oehrn C.R., Hanslmayr S., Fell J., Deuker L., Kremers N.A., Lam A.T.D., Elger C.E., Axmacher N. (2014). Neural communication patterns underlying conflict detection, resolution, and adaptation. J. Neurosci..

[bb0175] Pizzagalli D.A., Oakes T.R., Davidson R.J. (2003). Coupling of theta activity and glucose metabolism in the human rostral anterior cingulate cortex: an EEG/PET study of normal and depressed subjects. Psychophysiology.

[bb9010] Rabbitt P.M. (1966). Errors and error correction in choice-response tasks. J Exp Psychol..

[bb0180] Ratcliff R., Frank M.J. (2012). Reinforcement-based decision making in corticostriatal circuits: mutual constraints by neurocomputational and diffusion models. Neural Comput..

[bb0185] Ridderinkhof K.R., Ullsperger M., Crone E.A., Nieuwenhuis S. (2004). The role of the medial frontal cortex in cognitive control. Science.

[bb0190] Rodriguez-Oroz M.C., López-Azcárate J., Garcia-Garcia D., Alegre M., Toledo J., Valencia M., Guridi J., Artieda J., Obeso J.A. (2011). Involvement of the subthalamic nucleus in impulse control disorders associated with Parkinson's disease. Brain.

[bb0195] Rustamov N., Rodriguez-Raecke R., Timm L., Agrawal D., Dressler D., Schrader C., Tacik P., Wegner F., Dengler R., Wittfoth M., Kopp B. (2013). Absence of congruency sequence effects reveals neurocognitive inflexibility in Parkinson's disease. Neuropsychologia.

[bb0200] Sheth S.A., Mian M.K., Patel S.R., Asaad W.F., Williams Z.M., Dougherty D.D., Bush G., Eskandar E.N. (2012). Human dorsal anterior cingulate cortex neurons mediate ongoing behavioural adaptation. Nature.

[bb0205] Siegert S., Herrojo Ruiz M., Brücke C., Huebl J., Schneider G.-H., Ullsperger M., Kühn A.A. (2014). Error signals in the subthalamic nucleus are related to post-error slowing in patients with Parkinson's disease. Cortex J. Devoted Study Nerv. Syst. Behav.

[bb0210] Van Meel C.S., Heslenfeld D.J., Oosterlaan J., Sergeant J.A. (2007). Adaptive control deficits in attention-deficit/hyperactivity disorder (ADHD): the role of error processing. Psychiatry Res..

[bb0215] Wang C., Ulbert I., Schomer D.L., Marinkovic K., Halgren E. (2005). Responses of human anterior cingulate cortex Microdomains to error detection, conflict monitoring, stimulus–response mapping, familiarity, and orienting. J. Neurosci..

[bb0220] Zavala B., Brittain J.-S., Jenkinson N., Ashkan K., Foltynie T., Limousin P., Zrinzo L., Green A.L., Aziz T., Zaghloul K., Brown P. (2013). Subthalamic nucleus local field potential activity during the Eriksen flanker task reveals a novel role for theta phase during conflict monitoring. J. Neurosci..

[bb0225] Zavala B.A., Tan H., Little S., Ashkan K., Hariz M., Foltynie T., Zrinzo L., Zaghloul K.A., Brown P. (2014). Midline frontal cortex low-frequency activity drives subthalamic nucleus oscillations during conflict. J. Neurosci..

[bb0230] Zavala B., Damera S., Dong J.W., Lungu C., Brown P., Zaghloul K.A. (2015). Human subthalamic nucleus theta and beta oscillations entrain neuronal firing during sensorimotor conflict. Cereb. Cortex.

[bb0235] Zavala B., Zaghloul K., Brown P. (2015). The subthalamic nucleus, oscillations, and conflict. Mov. Disord..

[bb0240] Zavala B., Tan H., Little S., Ashkan K., Green A.L., Aziz T., Foltynie T., Zrinzo L., Zaghloul K., Brown P. (2016). Decisions made with less evidence involve higher levels of corticosubthalamic nucleus theta band synchrony. J. Cogn. Neurosci..

