# Supplementary material for: Human subthalamic nucleus–medial frontal cortex theta phase coherence is involved in conflict and error related cortical monitoring
Source: Neuroimage. 2016 Aug 15;137:178–87. doi: 10.1016/j.neuroimage.2016.05.031 (PMC4927260; doi:10.1016/j.neuroimage.2016.05.031)
Supplement: Supplementary file 1 — Supplementary figures. [file mmc1.docx]

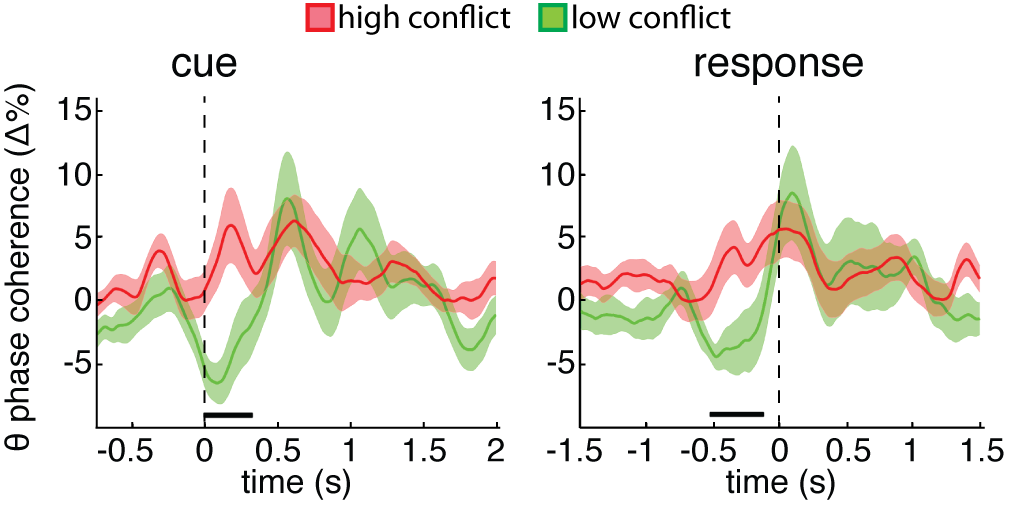


**Supplementary Figure 1: Subsampled STN-mesial frontal intersite theta phase coherence.** Same results from figure 2C, but these results were generated using only 6 trials in each condition to quantify the intersite phase coherence. This subsampled analysis was conducted to verify that 6 trials alone could be used to estimate the phase coherence between the two brain regions.


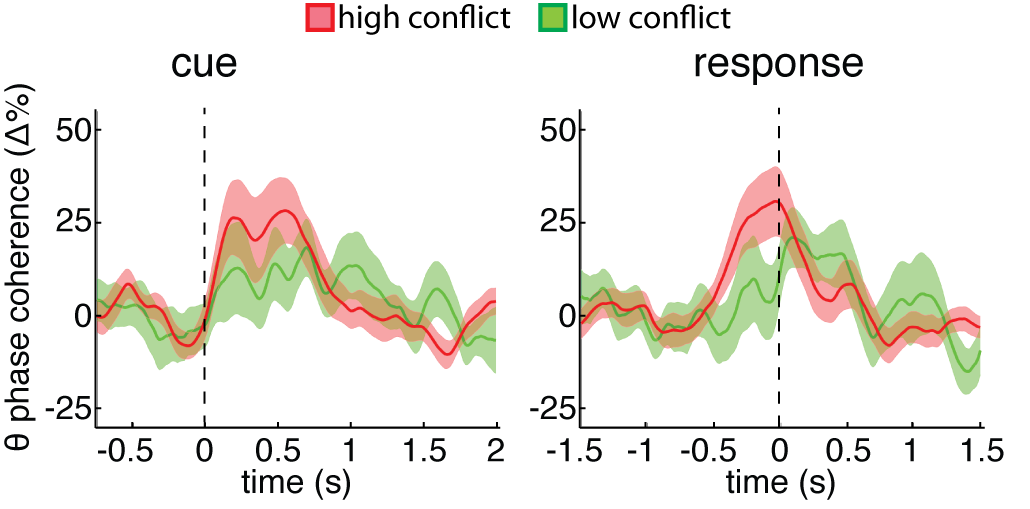


**Supplementary Figure 2: STN-parietal cortex intersite theta phase coherence.** Same as figure 2C, but the Pz-Cz electrode was used instead of the Fz-Cz electrode.
